# Practical Clinical Lessons on Syphilis and the Genito-Urinary Diseases

**Published:** 1886-07

**Authors:** 


**Practical Clinical Lessons on Syphilis and the Genito-Urinary Diseases** By FESSENDEN N. OTIS, M. D., Clinical Professor of Genito-Urinary Diseases, College Physicians and Surgeons, etc. New York: G. P. Putnam's Sons.

This is a cheap or students' edition of Otis' admirable work, which is so well known as to need no commendation. The author promises a second edition of the original work, with the addition of new material. This students' edition is published simply because the original book is practically out of print, and unobtainable, and the author finds that a considerable time must elapse before he can revise the old edition and prepare the proposed new matter for a second edition in a manner satisfactory to himself.

